# Molecular Epidemiology of Carbapenem Non-Susceptible *Acinetobacter baumannii* in France

**DOI:** 10.1371/journal.pone.0115452

**Published:** 2014-12-17

**Authors:** Katy Jeannot, Laure Diancourt, Sophie Vaux, Michelle Thouverez, Amandina Ribeiro, Bruno Coignard, Patrice Courvalin, Sylvain Brisse

**Affiliations:** 1 Institut Pasteur, Centre National de Référence de la Résistance aux Antibiotiques, Unité des Agents Antibactériens, Paris, France; 2 Institut Pasteur, Genotyping of Pathogens and Public Health, Paris, France; 3 French Institute for Public Health Surveillance (InVS), Saint-Maurice, France; 4 Laboratoire d'Epidémiologie et d'Hygiène Hospitalière, CHRU Jean Minjoz, Besançon, France; 5 Institut Pasteur, Microbial Evolutionary Genomics, Paris, France; 6 CNRS, UMR3525, Paris, France; University Medical Center Groningen, Netherlands

## Abstract

Carbapenem-resistant *Acinetobacter baumannii* have emerged globally. The objective of this study was to investigate the epidemiology, clonal diversity and resistance mechanisms of imipenem non-susceptible *A. baumannii* isolates in France. Between December 2010 and August 2011, 132 notifications were collected, including 37 outbreaks corresponding to 242 cases (2 to 55 per cluster). Multilocus sequence typing, pulsed-field gel electrophoresis (PFGE) and characterisation of carbapenemase-encoding genes were performed on 110 non-repetitive isolates. Gene *bla*_OXA-23_ was the most frequently detected (82%), followed by *bla*_OXA-24_ (11%) and *bla*_OXA-58_ (7%). Eleven sequence types (ST) were distinguished, among which sequence types ST1, ST2 (64%), ST20, ST25, ST85 and ST107. Isolates from epidemiological clusters had the same ST and resistance genes, indicating probable transmission within centres. In contrast, PFGE types of isolates differed among centres, arguing against transmission among centers. This study provides the first epidemiological snapshot of the population of *A. baumannii* with reduced susceptibility to carbapenems from France, and further underlines the predominance of international clones.

## Introduction

Carbapenem resistance in *Acinetobacter baumannii* is a significant health problem, particularly for critically ill patients in intensive care units, as it results in few antimicrobial treatment options [1], [2]. Acquired carbapenem resistance in this species is mainly associated with the production of carbapenemases [3], [4]. Most of these enzymes belong to the carbapenem-hydrolysing OXA-type class D ß-lactamase (CHDL). Six groups of CHDL genes have been identified in *A. baumannii*, namely the intrinsic chromosomal *bla*_OXA-51 like_ gene, and acquired genes *bla*_OXA-23 like_, *bla*_OXA-24 like_, *bla*_OXA-58 like_, *bla*_OXA-143 like_ and *_bla_*_OXA-235 like_ [4], [5]. Phylogenetically, *A. baumannii* is a homogeneous species, with most strains sharing >99.5% sequence similarity at housekeeping genes [6]. Pulsed-field gel electrophoresis is a widely used method to discriminate strains for epidemiological purposes [7]. Furthermore, clinical isolates of this species predominantly belong to a small number of widespread clones, including international clones I–III [8]–[10], also defined based on multilocus sequence typing (MLST) as clonal complexes CC1 to CC3 [6]. Although carbapenem resistance is associated with several clones and OXA enzymes in several countries, data from France are scarce [11]–[13] [14], [15] and no countrywide study has been performed. The goal of this study was to determine the epidemiology, clonal diversity and enzymes involved in reduced susceptibility to carbapenems in France.

## Materials and Methods

### Surveillance and epidemiological data

In France, notification of imipenem non-susceptible *A. baumannii* infections or colonisations is not mandatory but is especially recommended in case of outbreaks [16]. Notifications of imipenem non-susceptible isolates received by the French Institute for Public Health Surveillance (InVS) through a national Healthcare-Associated Infections Early Warning and Response System (HAI-EWRS) between December 2010 and August 2011 were reviewed; data on case numbers, type (infection or colonisation), clustering and type of healthcare facility were analyzed. A cluster was defined with two or more cases occurring in a time period and location where cross transmission was suspected.

### Bacterial isolates

A total of 110 carbapenem-non susceptible (MIC of imipenem >4 µg/mL; Clinical Laboratory Standards Institute [CLSI] breakpoint) *A. baumannii* clinical isolates were prospectively collected by the National Reference Centre (NRC) for Antimicrobial Resistance from 23 French laboratories affiliated to hospitals and clinics during the above study period. A single isolate per patient was included. Identification as *A. baumannii* was based on conventional techniques, automated instruments including Vitek-2 (bioMérieux, Marcy-l'Etoile, France) or mass spectrometry (Microflex, Bruker Corporation, Billerica MA; Vitek-MS, bioMérieux). Identification was confirmed by PCR detection of the naturally-occurring OXA-type ß-lactamase (OXA-51 and its variants) gene [17].

### Drug susceptibility determination

Susceptibility testing was performed by disk diffusion on Mueller-Hinton agar for piperacillin-tazobactam, ceftazidime, cefepime, imipenem, meropenem, ciprofloxacin, gentamicin and amikacin. MICs of meropenem and imipenem were determined by agar microdilution. Data were interpreted according to the Clinical Laboratory Standards Institute (CLSI) [18].

### PCR and sequencing of carbapenemase-encoding genes

A multiplex PCR assay was used to detect known OXA carbapenemase genes grouped into four sequence clusters *bla*_OXA-23-like_, *bla*_OXA-24/40- like_, *bla*_OXA-58-like_ and *bla*_OXA-51_ [19]. Detection of *bla*_OXA-143_ was performed by a separate PCR [20]. The presence of genes for Ambler class A (PER-, GES-, VEB) and B carbapenemases (IMP-, VIM-, NDM-) was investigated as described [21]. IS*Aba1* element upstream of the *bla*_OXA-51_ gene was searched for by PCR. Amplicons were sequenced on both strands on a 3100 DNA sequencer (PE Applied Biosystems) and sequences were compared with those in public databases.

### Genotyping

MLST was performed using the Institut Pasteur method [6]; http://www.pasteur.fr/mlst. Clonal complexes were defined as groups of sequence types (ST) differing by a single allelic mismatch with at least one other ST of the group. Pulsed-field gel electrophoresis (PFGE) with enzyme *Apa*I was achieved and interpreted according to Seifert et al. [7], with patterns showing six band differences or less among each other being considered as corresponding to the same type.

## Results

### Epidemiological data

**Figure 1:**
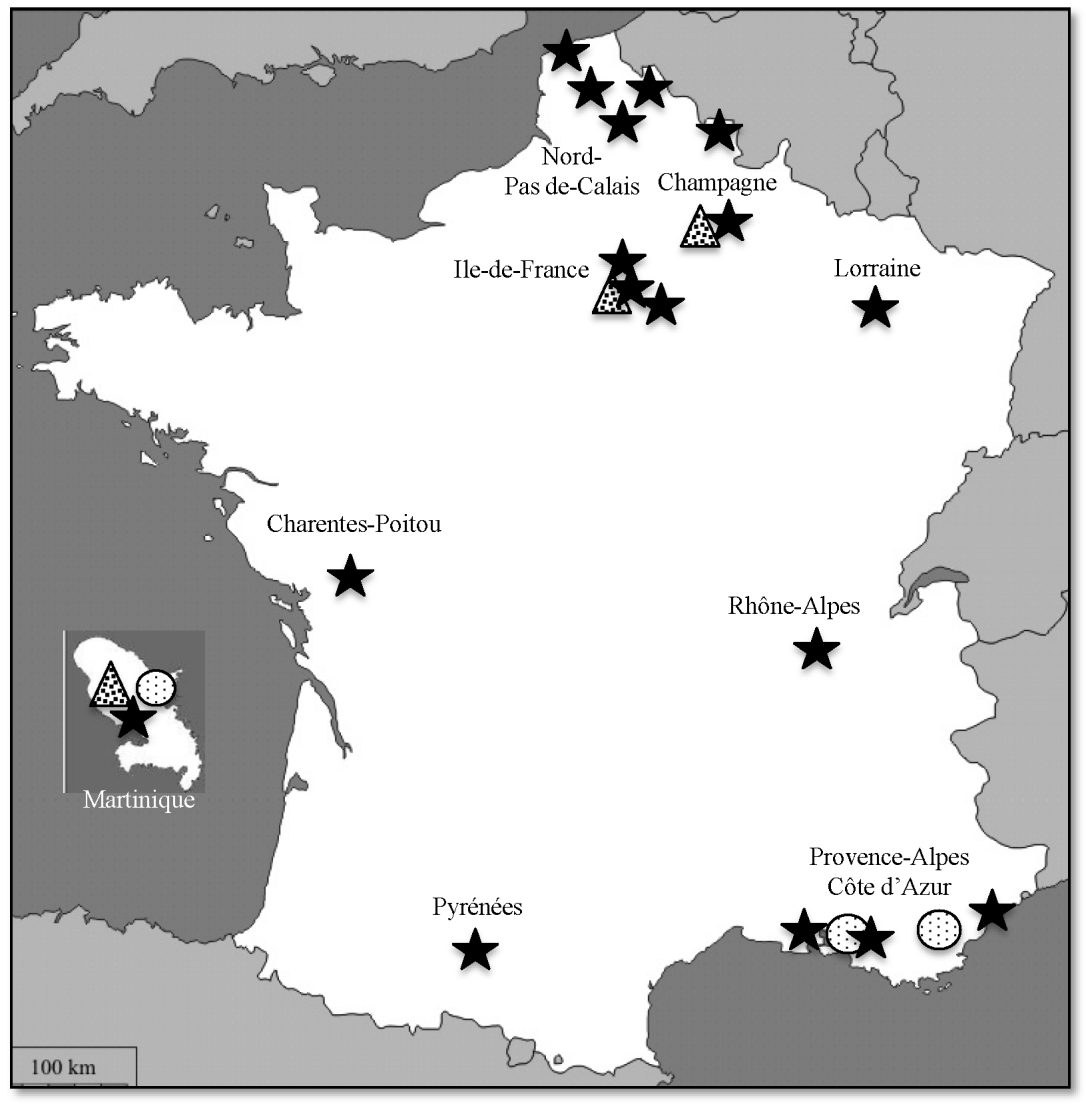
Geographical distribution of *A. baumannii* producing acquired oxacillinase enzymes. Carbapenem-hydrolyzing OXA-23, OXA-58 and OXA-24 are indicated by stars, circles and triangles, respectively.

Among 1,213 healthcare-associated infections notifications within the study period, 132 (10.9%) involved imipenem non-susceptible *A. baumannii* isolates, accounting for 283 cases. The 132 notifications included 37 clusters. The clusters accounted for 242 cases (2 to 55 per cluster). The median number of cases per cluster was 3 (mean: 6.5). Of 177 documented cases, 84 (47.5%) were infection and 93 (52.5%) were colonization. The most frequent infections were respiratory (34%), blood (19%) or cutaneous (19%) infections. The most frequent colonization sites were gastrointestinal (46%) or respiratory (36%). Among the 283 cases, 50 deaths were reported (crude mortality). The notifications were transmitted by 61 healthcare facilities (1 to 10 per facility), which were regional/university hospitals (41%), local public hospitals (34%), private hospitals (17%), long-term care facilities (5%) or cancer centres (3%). Of 153 documented wards, 81 (53%) were intensive care units, 32 (21%) were medical wards, 23 (15%) were chirurgical wards and 2 (1.5%) were burn care centers. Only four out of 27 French Regions accounted for 199 (71%) of the reported imipenem non-susceptible *A. baumannii* isolates: the Paris area (77 cases, 27%), Nord-Pas de Calais in Northern France (65 cases, 23%), Provence-Alpes-Côte d′Azur (31 cases, 11%) in Southern France and Martinique in the Caribbean (26 cases, 9%); these regions are indicated in Fig. 1.

### Resistance of *A. baumannii* isolates to carbapenems

From the 283 cases, 110 carbapenem-non susceptible isolates were referred to the NRC (**Table 1**). Ninety-seven (90%) isolates came from the four Regions listed above (**Fig. 1**). All isolates were intermediate or resistant (MIC, from 16 to 64 µg/mL) to imipenem and meropenem except three, which remained susceptible (MIC = 4 µg/mL) to meropenem (**Table 1**). No isolate was positive for genes encoding metallo-ß-lactamases IMP, VIM and NDM [15] or extended spectrum ß-lactamases (ESBL) GES and VEB. Ninety of the 110 strains (82%) harboured the *bla*_OXA-23_ gene, including one isolate 3400, which also presented the gene *bla*_PER-7_ encoding an ESBL with a weak carbapenemase activity. Genes *bla*_OXA-24_ and *bla*_OXA-58_ were detected in 12 (11%) and 8 (7%) isolates, respectively. Only two isolates carried both *bla*_OXA-23_ and *bla*_OXA-58_. Finally, imipenem resistance was associated in two isolates with overproduction of the intrinsic oxacillinase OXA-66.

**Table 1:** Epidemiological, phenotypic and genotypic features of the *A. baumannii* studied.

Strain ID	Region of isolation, healthcare centre (H) number	No. of strains^a^	PFGE type	ST	MIC (µg/mL)		Carbapenem resistance mechanism
					IMP	MEM	
3027	Lorraine	1	A	1	32	32	OXA-23
3041	Ile-de-France, H1	9	B	107	32	32	OXA-24
3048	Ile-de-France, H1	2	C	1	32	64	OXA-23
3073	Ile-de-France, H1	2	D	20	16	32	OXA-23
3144	Provence-Alpes-Côte d'Azur, H1	1	E	2	32	32	OXA-23
3145	Provence-Alpes-Côte d'Azur, H2	6	F	85	32	32	OXA-23
3150	Provence-Alpes-Côte d'Azur, H3	1	G^b^	2	32	32	OXA-23
3153	Provence-Alpes-Côte d'Azur, H3	1	G^b^	2	64	32	OXA-23
3167	Martinique	14	H	2	32	32	OXA-23
3169	Provence-Alpes-Côte d'Azur, H4	3	I	25	64	64	OXA-58
3171	Provence-Alpes-Côte d'Azur, H5	3	J	25	16	4	OXA-58
3182	Provence-Alpes-Côte d'Azur, H2	6	K^b^	2	32	32	OXA-23
3187	Provence-Alpes-Côte d'Azur, H6	1	K^b^	2	32	32	OXA-23
3189	Nord-Pas-de Calais, H1	21	L	2	32	32	OXA-23
3210	Poitou-Charentes	1	M	2	32	32	OXA-23
3211	Nord-Pas de Calais, H2	4	N^b^	2	32	16	OXA-23
3221	Martinique	2	O	79	64	64	OXA-23+ OXA-58
3222	Martinique	2	P	108	32	32	OXA-24
3270	Nord-Pas de Calais, H3	2	Q	1	64	64	OXA-23
3281	Nord-Pas de Calais, H4	3	R	115	16	32	OXA-23
3283	Nord-Pas de Calais, H4	4	S	2	32	32	OXA-23
3368	Provence-Alpes-Côte d'Azur, H7	1	T	2	32	32	OXA-23
3390	Midi-Pyrénées	1	U	2	64	64	OXA-23
3393	Rhône-Alpes	5	V	2	32	32	OXA-23
3395	Nord-Pas de Calais, H2	1	N^b^	2	32	32	OXA-23
3400	Champagne	1	W	25	64	64	OXA-23+ PER-7
3408	Nord-Pas de Calais, H5	1	X	10	32	16	OXA-23
3410	Ile-de-France, H2	5	Y	2	32	32	OXA-23
3415	Ile-de-France, H3	1	Z	2	32	16	OXA-23
3416	Champagne	1	AA	2	32	32	OXA-24
3435	Rhône-Alpes	1	AB	125	32	32	OXA-23
3458	Provence-Alpes-Côte d'Azur, H2	1	AC	20	32	32	OXA-23

PFGE, pulsed-field gel electrophoresis; MLST, Multilocus sequence typing; ST, sequence type;

IMP, imipenem; MEM, meropenem.

a Number of strains belonging to a single ST.

b These PFGE types were each observed in two distinct centres.

### Genotypic characterization

**Figure 2:**
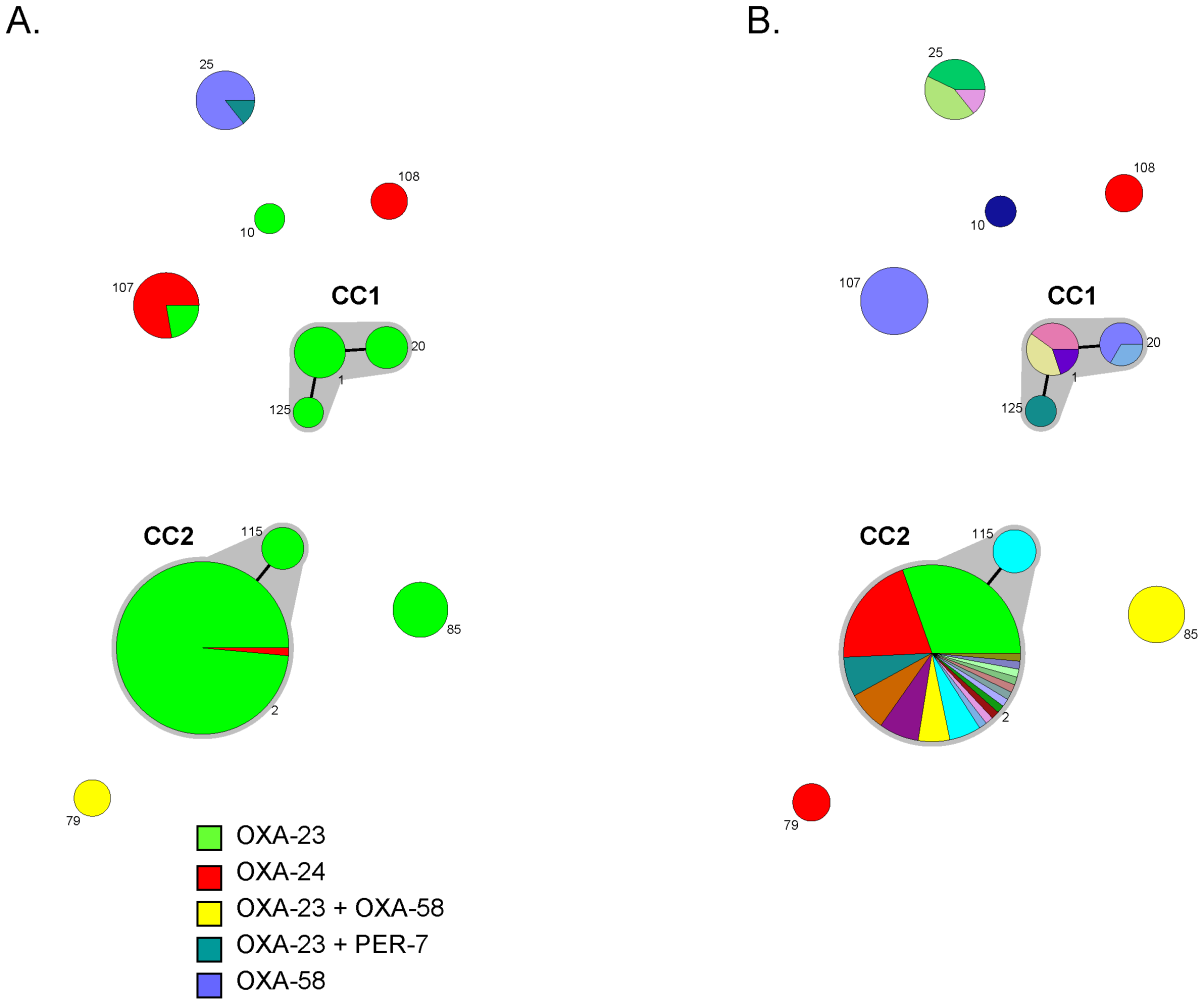
Genetic diversity of carbapenem-non susceptible *A. baumannii* from France. Each circle represents a sequence type (ST), which number is given besides the circle. The size of circles is related to the number of isolates. Grey zones between circles represent clonal complexes. The relative positioning of clonal complexes and singletons STs is not necessarily related to their genetic proximity. A: Coloured sectors inside circles denote the proportion of isolates with each enzyme or combination thereof, as indicated in the key. B: Coloured sectors inside circles denote the proportion of isolates from each healthcare centre (arbitrary colours, with no correspondence with those in Fig. 2A).

MLST analysis of the 108 strains producing an acquired oxacillinase enzyme showed that they belonged to 11 sequence types (ST; **Fig. 2** **;** **Table 1**). A high frequency of ST2 was observed, with 69 (64%) isolates. Besides, ST115 (3 isolates) differed by a single gene (*pyrG*) from ST2 (**Fig. 2**), thus belonging to CC2 [6]. Likewise, ST1, ST20 and ST125 formed CC1 (**Fig. 2**), with nine isolates. Distribution of STs across centres (**Fig. 2B**) showed that ST2 was recovered from 17 distinct centres located in all French Regions. ST1 and ST20 were also isolated in centres from several Regions. ST25 (7 isolates), ST85 (6 isolates) and ST107 (9 isolates) also included multiple isolates, with the two latter coming from a single centre. Conversely, the results showed that several individual centres contributed distinct STs (**Table 1**). These results show that the carbapenem non-susceptible population of *A. baumannii* in France is polyclonal, with a large distribution of several STs across French Regions.

To determine the contribution of clonal spread to French *A. baumannii* cases, 32 isolates, each representing a sporadic isolate or a single member of a given cluster, were analyzed by PFGE. Remarkably, there were only three pairs of undistinguishable isolates (**Table 1**); they belonged to ST2 and carried *bla*_OXA-23_. These results do not provide evidence for frequent cross-transmission among distinct centres in France, in sharp contrast with a large multicenter outbreak that occurred in 2003–2004 [27].

The distribution of carbapenemase genes across STs (**Fig. 2A**; **Table 1**) showed that *bla*_OXA-23_ was present in isolates of most STs. This gene was the only one found in CC1 and was largely dominant in CC2, with 68 out of 69 (98.6%) isolates harbouring this gene. The single exception was an isolate with *bla*_OXA-24_. In contrast, a majority of ST107 and ST25 isolates harboured *bla*_OXA-24_ and *bla*_OXA-58_, respectively. These results show that most isolates of a given ST produced the same carbapenem-hydrolyzing enzyme. This could be explained by acquisition from a common ancestor, multiple independent acquisitions and/or horizontal transfer among members of a given ST.

## Discussion

Although no national surveillance system is in place for imipenem-resistant *A. baumannii* strains in France, the French Healthcare-Associated Infections Early Warning and Response System (HAI-EWRS) proved operational for the rapid identification of clusters of such strains. However, it has to be noted that underreporting is likely given that notifications were not mandatory, and a dedicated survey would be necessary in order to assess the incidence or total number of cases occurring in France. However, data from the 2012 French Point Prevalence Survey [28] showed that *A. baumannii* infections are not frequent among French healthcare facilities, only accounting for a prevalence of 0.02% per 100 patients. Taking their rarity into account, our approach focusing on the rapid identification of major imipenem-resistant *Acinetobacter baumannii* outbreaks was deemed appropriate, as for other emerging pathogens such as vancomycin-resistant *Enterococcus* or 027 *Clostridium difficile* [29], [30]. Other countries might adopt other surveillance strategies based on their epidemiology. In order to better ascertain the European situation regarding multidrug-resistant *A. baumannii* infections, the European Center for Disease Prevention and Control (ECDC) recently added this pathogen in the EARS-Net surveillance scheme [31].

The notifications of imipenem-resistant *A. baumannii* strains received by the French Institute for Public Health Surveillance (InVS) and the carbapenem resistance mechanisms identified in *A. baumannii* strains by the NRC for Antibimicrobial Resistance testify for an increase of carbapenem-resistant isolates every year since 2009. More than 10% of the notifications on the study period were related with imipenem-resistant *A. baumannii* isolates. When taking in consideration historical data [32], analysis performed from 2001 to August 2011 showed that the proportion of these strains among all healthcare-associated infections notifications remained low from 2001 to 2002, increased in 2003 (2.6%), remained relatively stable between 2003 and 2009 (between 2.1 and 3.2%) and then increased dramatically during the following years (5.1% in 2009). Notifications of imipenem-resistant *A. baumannii* strains allowed identifying four regions where outbreaks of particular importance occurred during the study period: the Paris area, Nord-Pas-de-Calais, Provence-Alpes-Côtes-d′Azur Region and Martinique. In the Nord-Pas-de-Calais, a regional outbreak was ongoing since September 2010. Of note, one fifth of notifications were associated with cross-border exchange, with the most frequently reported countries being Algeria, Greece and Turkey (data not shown). The control of imipenem-resistant *A. baumannii* strains involved in outbreaks requires the implementation of very strict control measures around cases, including the reinforcement of standard and contact precautions, environmental cleaning, contact tracing and screening, or ward closure if needed.

In this study, the strains harbouring a carbapenem resistance mechanism were essentially recovered from respiratory samples from patients hospitalized in intensive-care-unit. As expected, the OXA-23 enzyme was the main contributor to imipenem non-susceptibility in France. In contrast, other enzymes such as bla_OXA-58_ or bla_OXA-51-like_ were predominant in Italy, Greece, Turkey, Lebanon, the Czech Republic [22], [33] and Japan [34]. However, OXA-23 was recently shown to increase in frequency in Italy and Greece [35]–[37] and in Germany, where it predominated in 2009 but not in 2005 and 2007 [38]. In a recent study of USA isolates, *bla*_OXA-23_ was also the most common gene [39]. These observations suggest an emergence of OXA-23. In Italy, the shift from OXA-58 to OXA-23 was associated with the expansion of particular subgroups of CC2 genotypes [37]. The STs found to be frequent in this survey were previously described among carbapenem non-susceptible isolates from France and other countries [2], [6], [14], [15], [22]–[26]. These results add to the growing evidence that the population of carbapenem non-susceptible *A. baumannii* isolates is genetically structured into a few international clones. In conclusion, we combined epidemiological and microbiological characterization of the population of carbapenem non-susceptible *A. baumannii* from France. The results demonstrate the predominance of *bla*_OXA-23_-like enzymes, consistent with recent trends in other countries. Although multiple clones were present, ST2 (international clone II) was by far the most frequent, consistent with the situation in many countries. The unique biological properties and epidemiology of successful international clones remain to be deciphered. Clusters of isolates were shown to involve identical ST and resistance genes, suggesting that clonal spread within centres played an important role. In contrast, there was very limited evidence of inter-centre transmission.

## References

[1] Peleg AY, Seifert H, Paterson DL (2008) *Acinetobacter baumannii*: emergence of a successful pathogen. Clin Microbiol Rev 21:538–582. 18625687 10.1128/CMR.00058-07 PMC2493088

[2] Gogou V, Pournaras S, Giannouli M, Voulgari E, Piperaki ET, et al (2011) Evolution of multidrug-resistant *Acinetobacter baumannii* clonal lineages: a 10 year study in Greece (2000-09). J Antimicrob Chemother 66:2767–2772. 21933784 10.1093/jac/dkr390

[3] Poirel L, Bonnin RA, Nordmann P (2011) Genetic basis of antibiotic resistance in pathogenic *Acinetobacter* species. IUBMB Life 63:1061–1067. 21990280 10.1002/iub.532

[4] Poirel L, Naas T, Nordmann P (2010) Diversity, epidemiology, and genetics of class D beta-lactamases. Antimicrob Agents Chemother 54:24–38. 19721065 10.1128/AAC.01512-08 PMC2798486

[5] Higgins PG, Perez-Llarena FJ, Zander E, Fernandez A, Bou G, et al (2013) OXA-235, a novel class D beta-lactamase involved in resistance to carbapenems in *Acinetobacter baumannii* . Antimicrob Agents Chemother 57:2121–2126. 23439638 10.1128/AAC.02413-12 PMC3632948

[6] Diancourt L, Passet V, Nemec A, Dijkshoorn L, Brisse S (2010) The population structure of *Acinetobacter baumannii*: expanding multiresistant clones from an ancestral susceptible genetic pool. PLoS One 5:e10034. 20383326 10.1371/journal.pone.0010034 PMC2850921

[7] Seifert H, Dolzani L, Bressan R, van der Reijden T, van Strijen B, et al (2005) Standardization and interlaboratory reproducibility assessment of pulsed-field gel electrophoresis-generated fingerprints of *Acinetobacter baumannii* . J Clin Microbiol 43:4328–4335. 16145073 10.1128/JCM.43.9.4328-4335.2005 PMC1234071

[8] Dijkshoorn L, Aucken H, Gerner-Smidt P, Janssen P, Kaufmann ME, et al (1996) Comparison of outbreak and nonoutbreak *Acinetobacter baumannii* strains by genotypic and phenotypic methods. J Clin Microbiol 34:1519–1525. 8735109 10.1128/jcm.34.6.1519-1525.1996 PMC229053

[9] van Dessel H, Dijkshoorn L, van der Reijden T, Bakker N, Paauw A, et al (2004) Identification of a new geographically widespread multiresistant *Acinetobacter baumannii* clone from European hospitals. Res Microbiol 155:105–112. 14990262 10.1016/j.resmic.2003.10.003

[10] Higgins PG, Dammhayn C, Hackel M, Seifert H (2009) Global spread of carbapenem-resistant *Acinetobacter baumannii* . J Antimicrob Chemother. 10.1093/jac/dkp428 19996144

[11] Tankovic J, Legrand P, De Gatines G, Chemineau V, Brun-Buisson C, et al (1994) Characterization of a hospital outbreak of imipenem-resistant *Acinetobacter baumannii* by phenotypic and genotypic typing methods. J Clin Microbiol 32:2677–2681. 7852555 10.1128/jcm.32.11.2677-2681.1994 PMC264141

[12] Heritier C, Dubouix A, Poirel L, Marty N, Nordmann P (2005) A nosocomial outbreak of *Acinetobacter baumannii* isolates expressing the carbapenem-hydrolysing oxacillinase OXA-58. J Antimicrob Chemother 55:115–118. 15590718 10.1093/jac/dkh500

[13] Kempf M, Rolain JM, Azza S, Diene S, Joly-Guillou ML, et al (2013) Investigation of *Acinetobacter baumannii* resistance to carbapenems in Marseille hospitals, south of France: a transition from an epidemic to an endemic situation. APMIS 121:64–71. 23030740 10.1111/j.1600-0463.2012.02935.x

[14] Bonnin RA, Cuzon G, Poirel L, Nordmann P (2013) Multidrug-resistant *Acinetobacter baumannii* clone, France. Emerg Infect Dis 19:822–823. 23697750 10.3201/eid1905.121618 PMC3647512

[15] Decousser JW, Jansen C, Nordmann P, Emirian A, Bonnin RA, et al (2013) Outbreak of NDM-1-producing *Acinetobacter baumannii* in France, January to May 2013. Euro Surveill 18. 10.2807/1560-7917.es2013.18.31.20547 23929226

[16] Group TRTRWG (2009) "RAISIN" - a national programme for early warning, investigation and surveillance of healthcare-associated infection in France. Eurosurveillance 14:1–8. 19941798

[17] Turton JF, Woodford N, Glover J, Yarde S, Kaufmann ME, et al (2006) Identification of *Acinetobacter baumannii* by detection of the blaOXA-51-like carbapenemase gene intrinsic to this species. J Clin Microbiol 44:2974–2976. 16891520 10.1128/JCM.01021-06 PMC1594603

[18] National_Committee_for_Clinical_Laboratory_Standards (2010) Performance standards for antimicrobiaol susceptibility testing: twentieth informational supplement. Clinical and Laboratory Standards Institute, Wayne, PA.

[19] Woodford N, Ellington MJ, Coelho JM, Turton JF, Ward ME, et al (2006) Multiplex PCR for genes encoding prevalent OXA carbapenemases in *Acinetobacter* spp. Int J Antimicrob Agents 27:351–353. 16564159 10.1016/j.ijantimicag.2006.01.004

[20] Higgins PG, Lehmann M, Seifert H (2010) Inclusion of OXA-143 primers in a multiplex polymerase chain reaction (PCR) for genes encoding prevalent OXA carbapenemases in *Acinetobacter* spp. Int J Antimicrob Agents 35:305. 10.1016/j.ijantimicag.2009.10.014 20022220

[21] Bonnin RA, Nordmann P, Poirel L (2013) Screening and deciphering antibiotic resistance in *Acinetobacter baumannii*: a state of the art. Expert Rev Anti Infect Ther 11:571–583. 23750729 10.1586/eri.13.38

[22] Di Popolo A, Giannouli M, Triassi M, Brisse S, Zarrilli R (2011) Molecular epidemiological investigation of multidrug-resistant *Acinetobacter baumannii* strains in four Mediterranean countries with a multilocus sequence typing scheme. Clin Microbiol Infect 17:197–201. 20456455 10.1111/j.1469-0691.2010.03254.x

[23] Alsultan AA, Evans BA, Elsayed EA, Al-Thawadi SI, Al-Taher AY, et al (2013) High frequency of carbapenem-resistant *Acinetobacter baumannii* in patients with diabetes mellitus in Saudi Arabia. J Med Microbiol 62:885–888. 23518655 10.1099/jmm.0.057216-0

[24] Koh TH, Tan TT, Khoo CT, Ng SY, Tan TY, et al (2012) *Acinetobacter calcoaceticus-Acinetobacter baumannii* complex species in clinical specimens in Singapore. Epidemiol Infect 140:535–538. 21733253 10.1017/S0950268811001129

[25] Zarrilli R, Pournaras S, Giannouli M, Tsakris A (2013) Global evolution of multidrug-resistant *Acinetobacter baumannii clonal* lineages. Int J Antimicrob Agents 41:11–19. 23127486 10.1016/j.ijantimicag.2012.09.008

[26] Karah N, Sundsfjord A, Towner K, Samuelsen O (2012) Insights into the global molecular epidemiology of carbapenem non-susceptible clones of *Acinetobacter baumannii* . Drug Resist Updat 15:237–247. 22841809 10.1016/j.drup.2012.06.001

[27] Naas T, Coignard B, Carbonne A, Blanckaert K, Bajolet O, et al (2006) VEB-1 Extended-spectrum beta-lactamase-producing *Acinetobacter baumannii*, France. Emerg Infect Dis 12:1214–1222. 16965700 10.3201/eid1208.051547 PMC3291215

[28] Anonymous (2013) Réseau d′alerte, d′investigation et de surveillance des infections nosocomiales (Raisin). Enquête nationale de prévalence des infections nosocomiales et des traitements anti-infectieux en établissements de santé, France, mai-juin 2012. Résultats. Saint-Maurice: InVS. 181 p.

[29] Bourdon N, Fines-Guyon M, Thiolet JM, Maugat S, Coignard B, et al (2011) Changing trends in vancomycin-resistant enterococci in French hospitals, 2001-08. J Antimicrob Chemother 66:713–721. 21393182 10.1093/jac/dkq524

[30] Coignard B, Barbut F, Blanckaert K, Thiolet JM, Poujol I, et al (2006) Emergence of *Clostridium difficile* toxinotype III, PCR-ribotype 027-associated disease, France, 2006. Euro Surveill 11:E060914 060911. 10.2807/esw.11.37.03044-en 17075146

[31] European Centre for Disease Prevention and Control. European Antimicrobial Resistance Surveillance Network (EARS-Net). Available: http://www.ecdc.europa.eu/en/activities/surveillance/EARS-Net/Pages/index.aspx

[32] Vaux S, Nguyen E, Alleaume S, Blanckaert K, Galas M, et al (2011) Signalement des infections nosocomiales à *Acinetobacter baumannii* résistant à l′imipénème, France, août 2001-mai 2011. Bulletin Epidémiologique Hebdomadaire 31–32:355–360.

[33] Nemec A, Krizova L, Maixnerova M, Diancourt L, van der Reijden TJ, et al (2008) Emergence of carbapenem resistance in *Acinetobacter baumannii* in the Czech Republic is associated with the spread of multidrug-resistant strains of European clone II. J Antimicrob Chemother 62:484–489. 18477708 10.1093/jac/dkn205

[34] Endo S, Yano H, Hirakata Y, Arai K, Kanamori H, et al (2012) Molecular epidemiology of carbapenem-non-susceptible *Acinetobacter baumannii* in Japan. J Antimicrob Chemother 67:1623–1626. 22447879 10.1093/jac/dks094

[35] D'Arezzo S, Principe L, Capone A, Petrosillo N, Petrucca A, et al (2011) Changing carbapenemase gene pattern in an epidemic multidrug-resistant *Acinetobacter baumannii* lineage causing multiple outbreaks in central Italy. J Antimicrob Chemother 66:54–61. 21088019 10.1093/jac/dkq407 PMC3031335

[36] Liakopoulos A, Miriagou V, Katsifas EA, Karagouni AD, Daikos GL, et al (2012) Identification of OXA-23-producing *Acinetobacter baumannii* in Greece, 2010 to 2011. Euro Surveill 17. 22449866

[37] Minandri F, D'Arezzo S, Antunes LC, Pourcel C, Principe L, et al (2012) Evidence of diversity among epidemiologically related carbapenemase-producing *Acinetobacter baumannii* strains belonging to international clonal lineage II. J Clin Microbiol 50:590–597. 22205821 10.1128/JCM.05555-11 PMC3295171

[38] Schleicher X, Higgins PG, Wisplinghoff H, Korber-Irrgang B, Kresken M, et al (2013) Molecular epidemiology of *Acinetobacter baumannii* and *Acinetobacter nosocomialis* in Germany over a 5-year period (2005-2009). Clin Microbiol Infect 19:737–742. 23034071 10.1111/1469-0691.12026

[39] Adams-Haduch JM, Onuoha EO, Bogdanovich T, Tian GB, Marschall J, et al (2011) Molecular epidemiology of carbapenem-nonsusceptible *Acinetobacter baumannii* in the United States. J Clin Microbiol 49:3849–3854. 21918019 10.1128/JCM.00619-11 PMC3209126

